# Psychological maltreatment and self-compassion - the mediating role of shame and perspective-taking

**DOI:** 10.1192/j.eurpsy.2022.227

**Published:** 2022-09-01

**Authors:** G. Vizin, H. Szőcs, Z. Illyés

**Affiliations:** 1 Semmelweis University, Department Of Clinical Psychology, Budapest, Hungary; 2 Eötvös Lorand University Institute of Psychology, Department Of Clinical Psychology And Addiction, Budapest, Hungary; 3 National Institute of Medical Rehabilitation, National Institute Of Medical Rehabilitation, Budapest, Hungary; 4 Centre of Cognitive and Schema Therapy, Centre Of Cognitive And Schema Therapy, Budapest, Hungary

**Keywords:** perspective-taking, psychological maltreatment, shame, self-compassion

## Abstract

**Introduction:**

Psychological maltreatment such as emotional abuse or neglect is a serious risk factor for poorer mental and somatic health outcomes in life. A higher rate of psychological maltreatment experienced in childhood is a predictor of aversive emotional states such as shame, and can negatively influence factors of mentalization such as perspective-taking capacity in adulthood. However, emotional abuse or neglect are also negative predictors of self-compassion.

**Objectives:**

The purpose of the study was to test two mediating models. We hypothesized, that reduced perspective-taking capacity, as well as higher levels of shame due to psychological maltreatment can be causally linked to lower levels of self-compassion.

**Methods:**

We collected data from 120 healthy subjects (mean age=29.46, SD = 7.55) from Hungary We used Experience of Shame Scale, Interpersonal Reactivity Index, Childhood Trauma Scale, and the Self-Compassion Scale in our cross-sectional questionnaire study.

**Results:**

Psychological maltreatment is a significant negative predictor of self-compassion (b=-0,712; p<0.05), and shame seems to play a mediating role in this relationship (effect size= 0.231; p<0.05). Psychological maltreatment was not a statistically significant predictor of perspective-taking.

**Conclusions:**

Our results highlight that shame has a central role between childhood traumatization and psychological well-being. In the case of early emotional maltreatment we have to focus on shame for higher levels of self-compassion and effective healing in psychotherapy.

**Disclosure:**

No significant relationships.

